# Imaging finding and arthroscopic treatment of isolated contracture of the rectus femoris muscle: a case report

**DOI:** 10.1186/s12891-019-2696-8

**Published:** 2019-07-29

**Authors:** Yunfeng Zhou, Zhengzheng Zhang, Jingyi Hou, Rui Yang

**Affiliations:** Department of Orthopaedics, Sun Yat-sen Memorial Hospital, Sun Yat-sen University, NO.107, Western Yanjiang Road, Yuexiu district, Guangzhou, Guangdong Province 510120 People’s Republic of China

**Keywords:** Knee flexion disorder, Muscle contracture, Fibrosis, Magnetic resonance imaging, Arthroscopy

## Abstract

**Background:**

Isolated rectus femoris (RF) contracture is encountered very rarely in orthopaedic practices. There are few reports on its imaging manifestations and no cases reported to be treated with arthroscopy.

**Case presentation:**

A 11-year-old girl with a more than 7 years history of restricted left knee flexion was presented. The clinical assessment and magnetic resonance imaging (MRI) findings were detailed here. A strip-like induration was palpated in the left thigh, which tends to be more obvious with knee flexion. MRI demonstrated a hypointensity band connected the anterior inferior iliac spine with the patella, and marked atrophy of the left RF muscle. Fibrosis contracture band was confirmed with arthroscope, then divided by radiofrequency ablation (RFA) under arthroscopic observation. Followed by debridement of the fibrillar connective tissue and hemostasis around the broken ends. The movement of left knee joint significantly improved after the operation, and the patient recovered nearly full range of motion of this joint after 6 months.

**Conclusion:**

The specific MRI findings could assist in confirming clinical early diagnosis of isolated RF contracture. Arthroscopic RFA treatment is an effective technique to treat this disorder with minimally incision.

**Electronic supplementary material:**

The online version of this article (10.1186/s12891-019-2696-8) contains supplementary material, which is available to authorized users.

## Background

Isolated contracture of RF muscle is a quite rare disease in clinics. Congenital factor, trauma, spasticity, and iatrogenic intramuscular injection in infancy or childhood might be the possible causes [1–3]. The disorder is more common in children, who are sent to clinics or hospitals for their abnormal walking pattern by their parents. Physical examination was the main basis to diagnose this disease in the early years, and its characteristic finding was that the knee couldn’t be flexed or flexion restricted when extended hip in the prone position, while in the supine position, the motion of the hip and knee was unlimited [4]. Lengthening of the quadriceps and distal transposition of the origin of the RF muscle through open avenues were the main surgical methods in traditional therapies. Herein, we report a case of isolated RF muscle contracture and describe the new findings on MRI manifestations in detail. Besides, we creatively attempt to treat this disorder with RFA under arthroscopy, obtaining satisfactory therapeutic effects.

## Case presentation

**Fig. 1:**
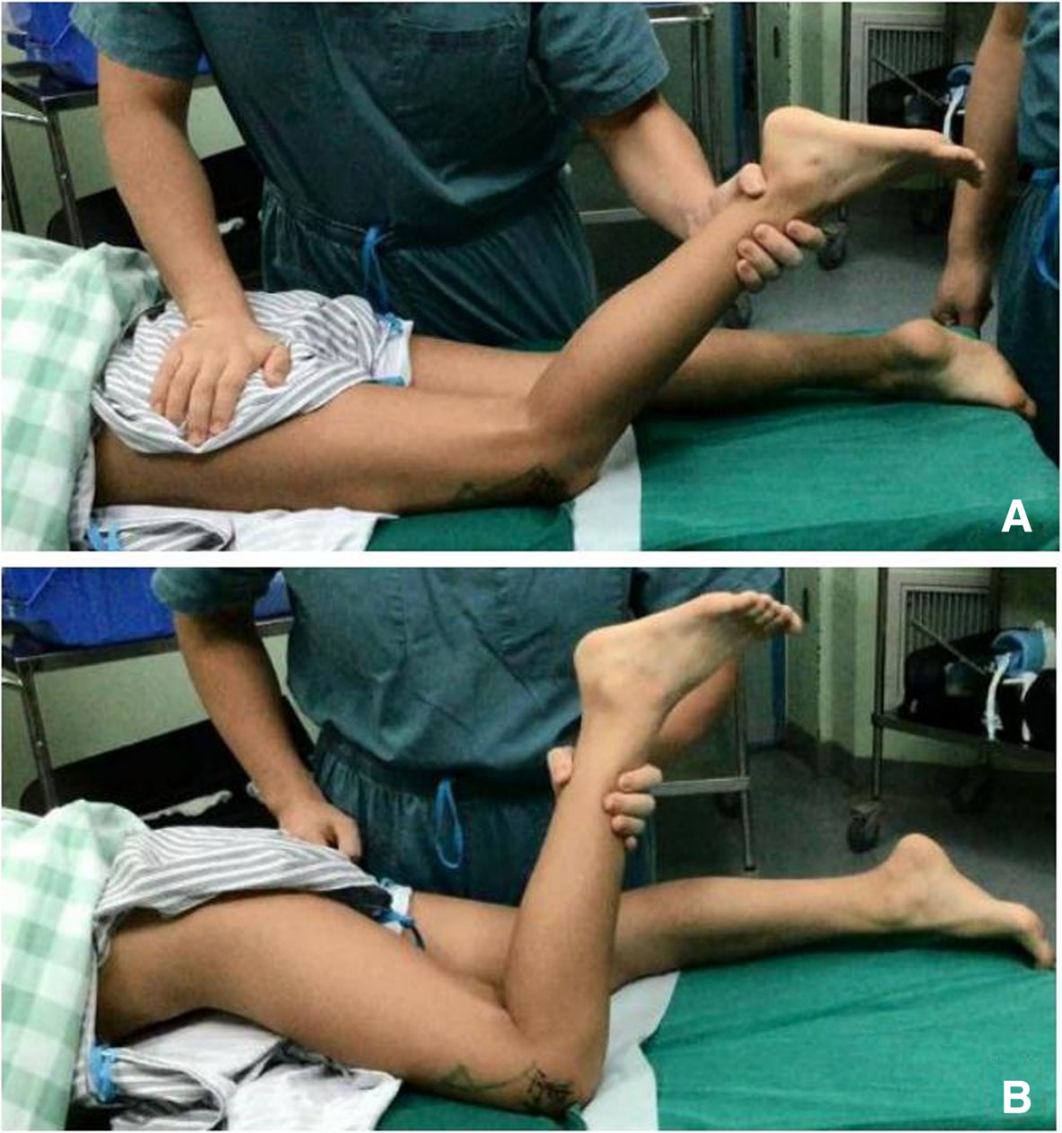
Preoperative physical examination. When the hip was fixed, knee flexion was limited (**a**). When the knee was forced to flex beyond the limitation, the hip was forced to flex simultaneously (**b**)

A 11-year-old girl complained that her left knee flexion was restricted when lying on bed in prone position. Her mother told us that her left gait pattern was mild abnormal since she began to walk. It was not treated and gradually progressed. She denied any knee trauma or pain since she could remember something. This little girl was full-term normal delivery without any physical activity disorder, and her parents had no problems. They declared that no intramuscular injection in her left thigh. Our examination revealed that her left lower limb slightly abducted during walking, especially in the initial period of moving the left foot forward. The movement of the knee free in the supine position with the hip flexion, the range of motion of the knee was measured from 0° to more than 135°. Whereas it was changed from 0° to about 50° when the hip was extended in the prone position. When the knee was forced for flexion, the ipsilateral hip was flexed spontaneously (Fig. 1). Besides, sitting in the Japanese-style showed an abnormal appearance with torso bended forward and the hips could not contact with the heels. A strip-like hardened band was palpated on the surface of left quadriceps, which became more obvious when the knee flexed while hip extended.

**Fig. 2:**
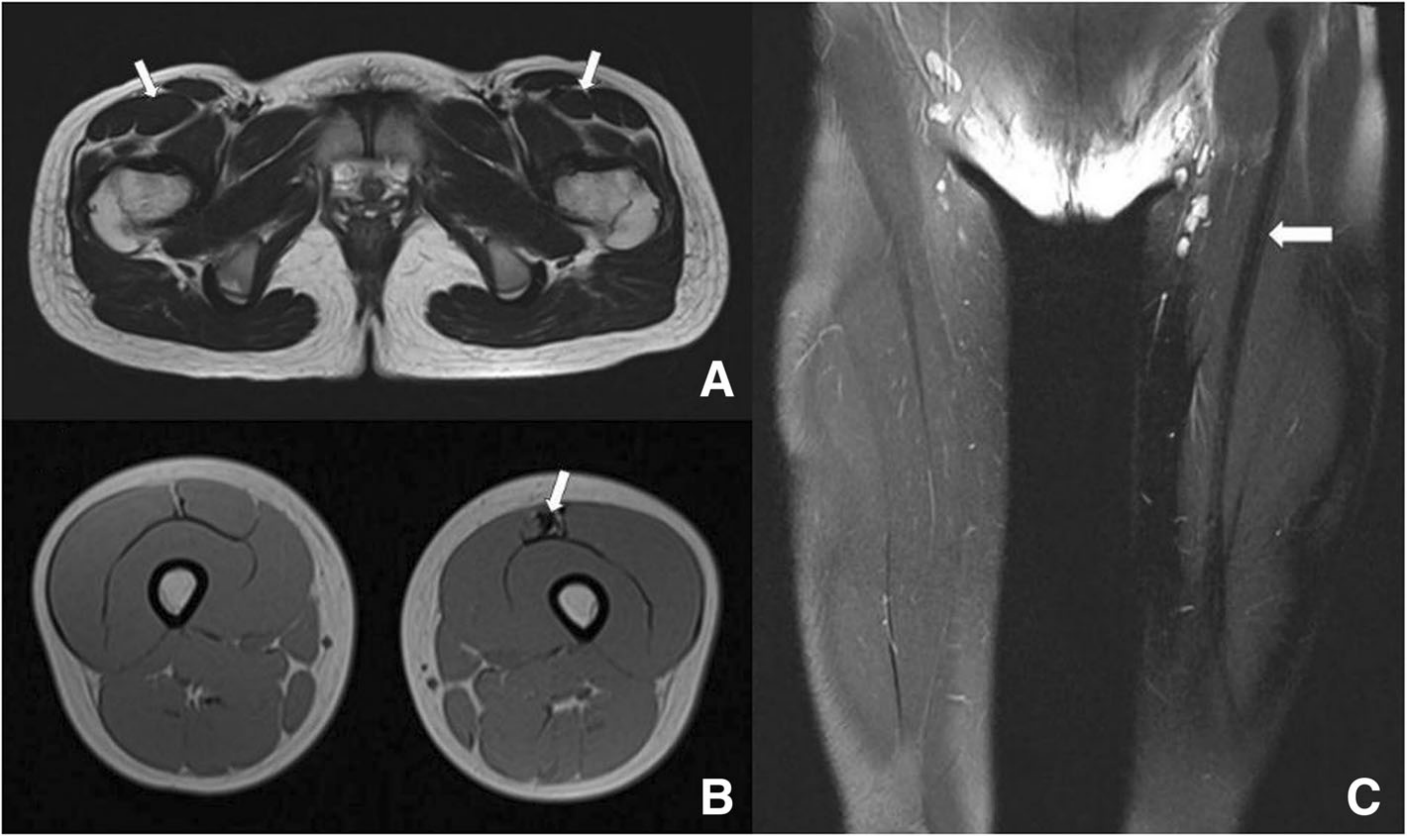
Preoperative MRI findings. Transverse T2-weighted image (**a**) showing volume reduction of left rectus femoris muscle. Transverse T1-weighted image (**b**) demonstrating fibrosis with dark signal intensity in the anterior of left rectus femoris. A hypointensity band could be observed on the coronal section (**c**) in the image. *White arrow in A*: rectus femoris muscle; *White arrow in B &C*: hypointensity band on the left rectus femoris muscle

MRI (Fig. 2) indicated that muscle volume of the left RF was smaller than the right one, and dark-signal intensity was detected inside the left RF muscle in transverse T1-weighted and T2-weighted images. By comparison with other muscles in the ipsilateral thigh or contralateral RF, the unnormal signal intensity suggest atrophy of the RF on the affected side. On the coronal sections, a hypointensity band connected the anterior inferior iliac spine with the patella was legible. The imaging findings implied that degeneration occurred in the RF muscle, or the latter was displaced by the fibrosis.

**Fig. 3:**
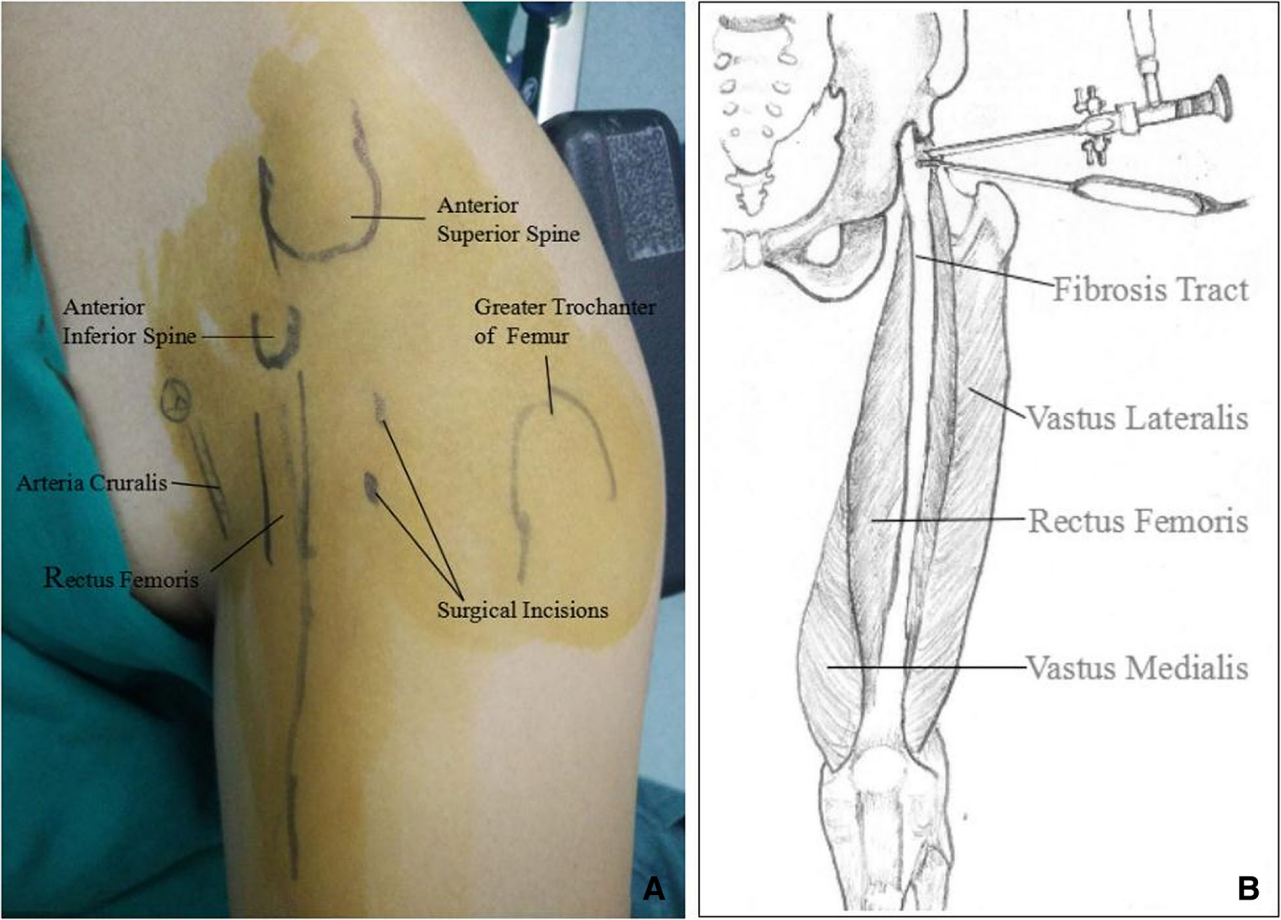
Body surface symbols and surgical incisions were marked before the operation (**a**) and the schematic diagram of the operation (**b**)

**Fig. 4:**
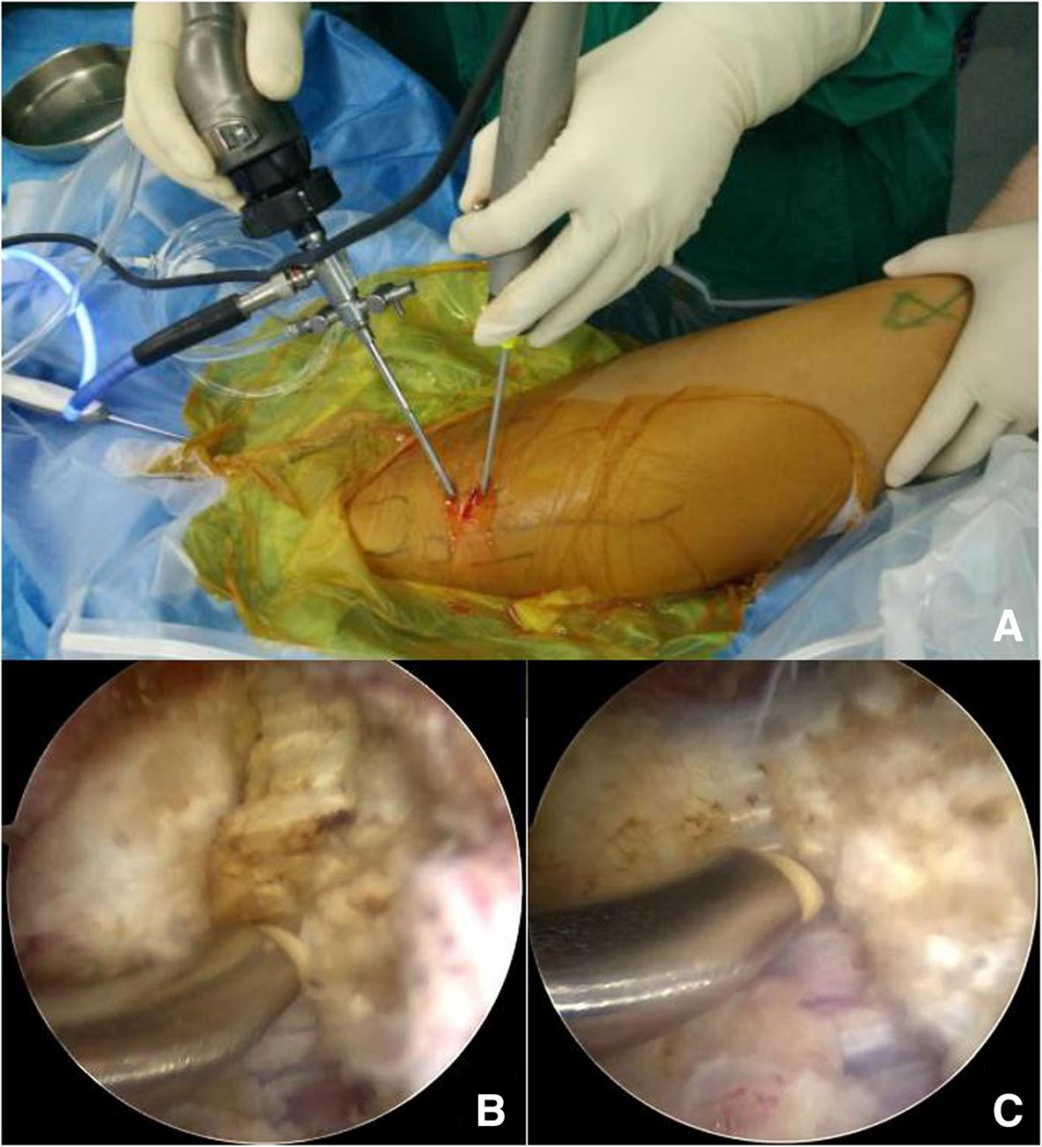
Gross appearance (**a**) and arthroscopic images (**b** & **c**) during the operation. Contracture band was divided by the ablation electrode gradually

Before the operation, body surface symbols and surgical incisions were marked (Fig. 3). A 70 degrees arthroscope (Arthrex Inc., Naples, Florida) with a diameter of 2.7 mm was used to visualize tissue via the proximal portal. RFA was performed through the distal approach and contracture band was divided by during arthroscopic procedure while the patient was under subarachnoid anesthesia in the right side-lying position with rigid buttocks fixation. This position facilitated the assessment of surgical effects during the operation by comparing the degree of knee flexion when the hip extended with that before the procedure. In the arthroscopic images, a thick contracture tract was discernible while the surrounding fibrous connective tissue was debrided by mechanical gouging system (Arthrex Inc., Naples, Florida) (Fig. 4a). Then a plasma knife was used to cut off the tract from the outside edge to the inside gradually (Fig. 4b & c). After complete transection, the passive knee flexion could easily reach 120°. Thorough debridement and hemostasis around the broken ends were conducted with the radiofrequency ablation.

The operation lasted 14 min. Two incisions about 0.6 cm for each in length were sutured and dressed. No cast or any other supporting instruments were used and the patient was placed in active position after anesthesia. The flexion angle of the left knee could easily reach more than 90° in the prone position with the hip extended on the first postoperative day. Active exercise was started 2 days after her surgery, for the pain in the surgical site was phenomenal on the first day. No symptom of neurovascular damage after surgery. During the postoperative examination, we found her myodynamia differences between operation side and the contralateral side were nonsignificant, neither between the lower limbs’ nor the thighs’.

**Fig. 5:**
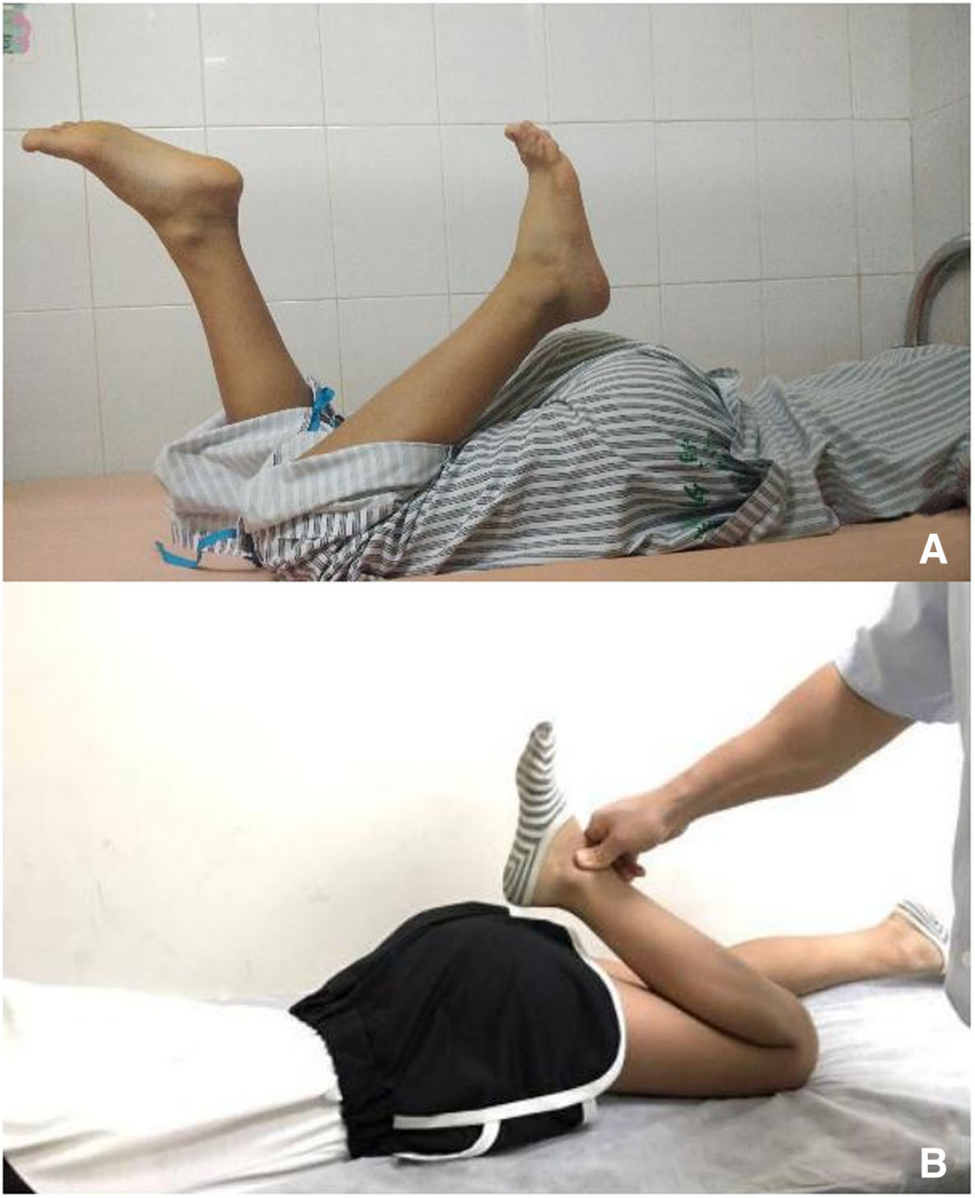
The flexion of left knee was obviously limited in the prone position (**a**) before the operation. But it reached nearly normal without pelvic elevation at 6-month follow-up (**b**)

At the 6-month follow-up, the patient had no pain, and nearly normal range of motion of the knee flexion (Fig. 5). She could walk and fell to her knees in a normal pattern, and she had returned to normal study and daily life. The girl and her families agreed that information about this case to be reported.

## Discussion and Conclusions

It has been reported that quadriceps contractures were caused by either congenital [3] or acquired factors, like trauma or intramuscular injection [1, 2, 5]. Ad Hoc Committee of the Japanese Orthopaedic Association had classified the contracture into three types: vastus form, rectus femoris form and combined form [6]. The RF is the only quadriceps muscle that connects the knee to the hip. Its direct and reflected heads originate from anterior inferior iliac spine and superolateral acetabular rim respectively [7]. Movements of the hip and knee in the supine position are free, while in the prone position, the knee flexion is limited or cannot be flexed [4]. The clinical findings manifest that the case belongs to the second style of contractures. But the etiology and pathogenesis of this case are still not clear.

The MRI findings revealed in the congenital contracture cases were first to reported by Nozawa [8]. In the literature, rectus femoris was reported to be replaced by fibrosis. The MR images of this case are consistent with the findings in published literatures. What’s more, we have detected a ribbon-like low signal intensity between the anterior inferior iliac spine and the superior border of patella in the coronal planes, which has some diagnostic values for the contracture that has never been reported previously. Besides, fibrosis was discovered upon the muscle under arthroscope, confirming the consistency of clinical checkup and radiological assessment.

Sasaki et al. [9] evaluated postoperative effects of different procedures to treat quadriceps contracture, they found that the pelvic elevation in the group with fibrosis release was higher than that in the group of pelvic release and sartorius plasty. The group treated by release of fibrosis achieved most satisfactory results and it was considered to be the best procedure to treat rectus style of quadriceps. Other solutions include elongation of the tendon of the quadriceps and distal transposition of the origin of the RF muscle. The tendon elongation might diminish its muscle strength, and the distal transposition would avoid strength weakening [4]. Transverse division of the RF at the muscle belly was recommended as the standard method and its published therapeutic effects were most satisfactory [3, 10]. Csink et al. [1] introduced a method that was dividing the muscle close to its origin with open surgery to treat this kind of disease. Along with the contracture was disconnected, the knee gained nearly normal function of flexion.

RFA induces resistive heating in tissue in direct connect with an ablation electrode [11]. The heat effect is the mainly therapeutic mechanism of ablation and cutting function. Its instruments incorporated with the use of a plasma bubble to ablate tissue with the aim of more efficient diathermy and reduction of temperature transmission to surrounding tissue [12]. In the authors’ opinion, building surgical approaches close to the RF muscle origin would be more propitious to expose the band or muscle and more beneficial to debridement and hemostasis. RF muscle flap are clinically regularly used to treat complex groin wounds [13]. In these procedures, the distal patellar attachment is disconnected and the muscle is brought into the groin wound beneath the subcutaneous tunnel. Most researchers have reported similar strength recovery, knee extension power and daily functionality after the use of flap. And no patient complained about operative limb weakness or instability [14]. We believe that merely dividing the contracture band near the proximal origin would not affect the myodynamia or adjacent joints movement. In fact, we haven’t discovered any significant differences of muscle strength at the 6-month follow-up.

The arthroscopic RFA treatment has been performed through two small portals and completed within 14 min. Similar to minimally invasive surgery, small trauma, short operation time and quick recovery are the main strengths of this operation. Moreover, radiofrequency knife also has hemostatic function, which is beneficial to reduce postoperative bleeding and re-adhesion. But it should be also kept in mind that the operation performed at the extrarticular region without a native cavity, the structure of tissue structure is not as obvious as it is in the joints. Therefore, it is imperative to master the anatomy and structure preoperatively. In addition, intraoperative water may flow into the surrounding interstitial spaces, which may induce water intoxication. Various effective measures could be taken to prevent the adverse outcome, such as being familiar with the structure, designing the operation plan in advance, shortening the operation time and avoiding the damage to normal tissues.

The case demonstrates the role of MRI in confirming the clinical early diagnosis of isolated contracture of RF muscle, and the minimally invasive treatment with RFA under arthroscope is feasible. The minimal invasive approach has the advantage of rapid recovery and brief hospitalization. What’s more, this case may explicit an alternative reference for the management of extra-articular disorder or diseases with intra-articular instruments, providing an example for the innovation of clinical treatment methods in clinics.


**Additional file 1:** Video S1. The physical examinations, MRI findings and postoperative clinical presentation of the case, as well as main surgical procedures under arthroscopy are showed in the video. (AVI 52607 kb)


## Data Availability

All data generated or analyzed during the current study are included in this article.

## Abbreviations

MRI: Magnetic resonance imaging
RF: Rectus femoris
RFA: Radiofrequency ablation
